# Anti-lipopolysaccharide factor D from kuruma shrimp exhibits antiviral activity

**DOI:** 10.1007/s42995-021-00113-y

**Published:** 2021-07-13

**Authors:** Hai-Shan Jiang, Li-Xia Lv, Jin-Xing Wang

**Affiliations:** Shandong Provincial Key Laboratory of Animal Cell and Developmental Biology, School of Life Sciences, Shandong University, Qingdao, 266237 China

**Keywords:** Anti-lipopolysaccharide factors, Cationic and anionic antimicrobial peptides, White spot syndrome virus, Viral envelope protein

## Abstract

Anti-lipopolysaccharide factors (ALFs) exhibit a potent antimicrobial activity against a broad range of bacteria, filamentous fungi, and viruses. In previous reports, seven groups of ALFs (groups A–G) were identified in penaeid shrimp. Among them, group D showed negative net charges and weak antimicrobial activity. Whether this group has antiviral function is not clear. In this study, the ALF sequences of penaeid shrimp were analyzed, and eight groups of ALF family (groups A–H) were identified. The four ALFs including MjALF-C2, MjALF-D1, MjALF-D2, and MjALF-E2 from kuruma shrimp *Marsupenaeus japonicus* were expressed recombinantly in *Escherichia coli*, and the antiviral activity was screened via injection of purified recombinant ALFs into shrimp following white spot syndrome virus (WSSV) infection. Results showed that the expression of *Vp28* (WSSV envelope protein) decreased significantly in the MjALF-D2-injected shrimp only. Therefore, MjALF-D2 was chosen for further study. Expression pattern analysis showed that *MjAlf-D2* was upregulated in shrimp challenged by WSSV. The WSSV replication was detected in RNA, genomic DNA, and protein levels using VP28 and *Ie1* (immediate-early gene of WSSV) as indicators in MjALF-D2-injected shrimp following WSSV infection. Results showed that WSSV replication was significantly inhibited compared with that in the rTRX- or PBS-injected control groups. After knockdown of *MjAlf-D2* in shrimp by RNA interference, the WSSV replication increased significantly in the shrimp. All these results suggested that MjALF-D2 has an antiviral function in shrimp immunity, and the recombinant ALF-D2 has a potential application for viral disease control in shrimp aquaculture.

## Introduction

White spot syndrome (WSS) in crustaceans is caused by WSS virus (WSSV), which is an enveloped circular double-stranded DNA virus, and it is a severely infectious disease that results in 100% mortality of shrimp within 3–10 days after infection (Peng et al. 2001). WSSV is a virulent pathogen not only to shrimp but also to wider crustacean species. Understanding the molecular mechanisms of the shrimp immune response against viral infection could provide new strategies for the disease control. Therefore, several studies were carried out, and great progress was achieved with shrimp immunity, especially in the discovery of antiviral effectors (Wang et al. 2014a; Zheng et al. 2019). Among them, antimicrobial peptides (AMPs), as the effector molecules, play important roles in shrimp antiviral immunity.

Several kinds of AMPs have been identified in shrimp, including penaeidins, crustins, anti-lipopolysaccharide factors (ALFs), and stylicins (Tassanakajon et al. 2011, 2018). Most of the peptides with net-positive charges possess antibacterial activity against Gram-positive or -negative bacteria, fungi, and viruses. ALF was first reported in chelicerates (horseshoe crab, *Limulus polyphemus*) as a potent anticoagulant factor (Morita et al. 1985; Ohashi et al. 1984; Tanaka et al. 1982). Since then, ALFs were identified in various crustaceans, such as portunid crabs (Afsal et al. 2012), freshwater prawn (Ren et al. 2012), crayfish (Jiravanichpaisal et al. 2007; Sun et al. 2011) and penaeid shrimp, such as *Litopenaeus vannamei*, *Fenneropenaeus chinensis, Penaeus monodon* and *M*. *japonicus* (Tassanakajon et al. 2018). Besides a conserved signal peptide, the sequences of mature ALF peptides contain a lipopolysaccharide (LPS)-binding domain formed between two conserved cysteine residues. The three-dimensional structure of ALFs consists of three α-helices and four stranded β-sheets (Yang et al. 2009). A central β-hairpin structure formed by 20 amino acid residues between two cysteines is stabilized by a disulfide bond. The central β-hairpin structure is the ALF functional domain which is involved in the recognition and binding of components from microbial cell walls (Schmitt et al. 2016).

The ALFs from penaeid shrimp constitute a diverse and multigenic family of AMPs. They are firstly divided into three groups (groups A–C) on the basis of sequence similarities and phylogenetic analysis (Tassanakajon et al. 2011). Group A consists of anionic and cationic polypeptides, whereas groups B and C are composed of highly cationic polypeptides. Subsequently, group D shrimp ALFs with negative net charges are identified and display impaired LPS-binding activity, and weak antimicrobial activity (Rosa et al. 2013). Then, a new group of ALFs, including MjALF-E1 and -E2 (designated as group E), was identified from *Marsupenaeus japonicus* (Jiang et al. 2015); they exhibit antimicrobial activity against bacteria. In addition, two novel groups of the family (groups F and G) were recently identified in *L*. *vannamei* (Matos et al. 2018). Group F is composed of cationic peptides with antibacterial and antifungal activities, whereas the antimicrobial activities of group G belonging to anionic peptides have not been identified (Matos et al. 2018). The ALFs with net positively charged LPS-Binding Domain (such as groups B, C, and F), which is highly conserved in sequence of each group, exhibit high antimicrobial activity, and anionic ALFs (such as group D) have weak or no antimicrobial activity. The seven ALFs could be simultaneously identified in a single shrimp, such as *L. vannamei* and *M. japonicus* (Jiang et al. 2015; Matos et al. 2018), possibly suggesting that different ALFs may act synergistically to improve their antimicrobial activity in vivo.

Functional analyses showed that ALF proteins exhibit a potent antimicrobial activity against a wide range of micro-organisms, including Gram-positive and Gram-negative bacteria and filamentous fungi (Rosa et al. 2013). ALFs also play an important role in the defense against viral pathogens in crustaceans. In vivo function analysis in crayfish *Pacifastacus leniusculus* showed that the knockdown expression of the ALF gene by double-stranded RNA (dsRNA) injection enhanced the expression of VP28, the envelope protein of WSSV (Liu et al. 2006). Silencing the LvALF in *L. vannamei* by RNA inference could also increase mortality after WSSV infection (de la Vega et al. 2008). The synthetic LBD (LPS-binding domain) of *Fc*ALF and *Fc*ALF2 (group B) from the shrimp *F. chinensis* could inhibit the replication of WSSV in vivo (Li et al. 2014; Liu et al. 2005). The ALFPm3 (group C) identified in *Penaeus monodon* exhibits anti-WSSV function by binding to the target structural protein complex of WSSV and leading to virion damage (Methatham et al. 2017).

Previous studies identified seven groups of ALFs (groups A–G) in kuruma shrimp *M. japonicus*, and the antibacterial functions of some of these ALFs were studied (Jiang et al. 2015). The anionic ALF-Ds showed weak antimicrobial activity (Rosa et al. 2013). Whether the anionic ALFs also has antiviral activity is not clear. In the present study, the phylogenetic tree of ALFs from penaeid shrimp was reconstructed, and a new group of ALF family (group H) was identified. The function of anionic MjALF-D2 in shrimp immunity was analyzed, and the anti-WSSV activity of ALF was authenticated in kuruma shrimp.

## Results

### Eight groups of the ALF family were identified in penaeid shrimp

**Fig. 1:**
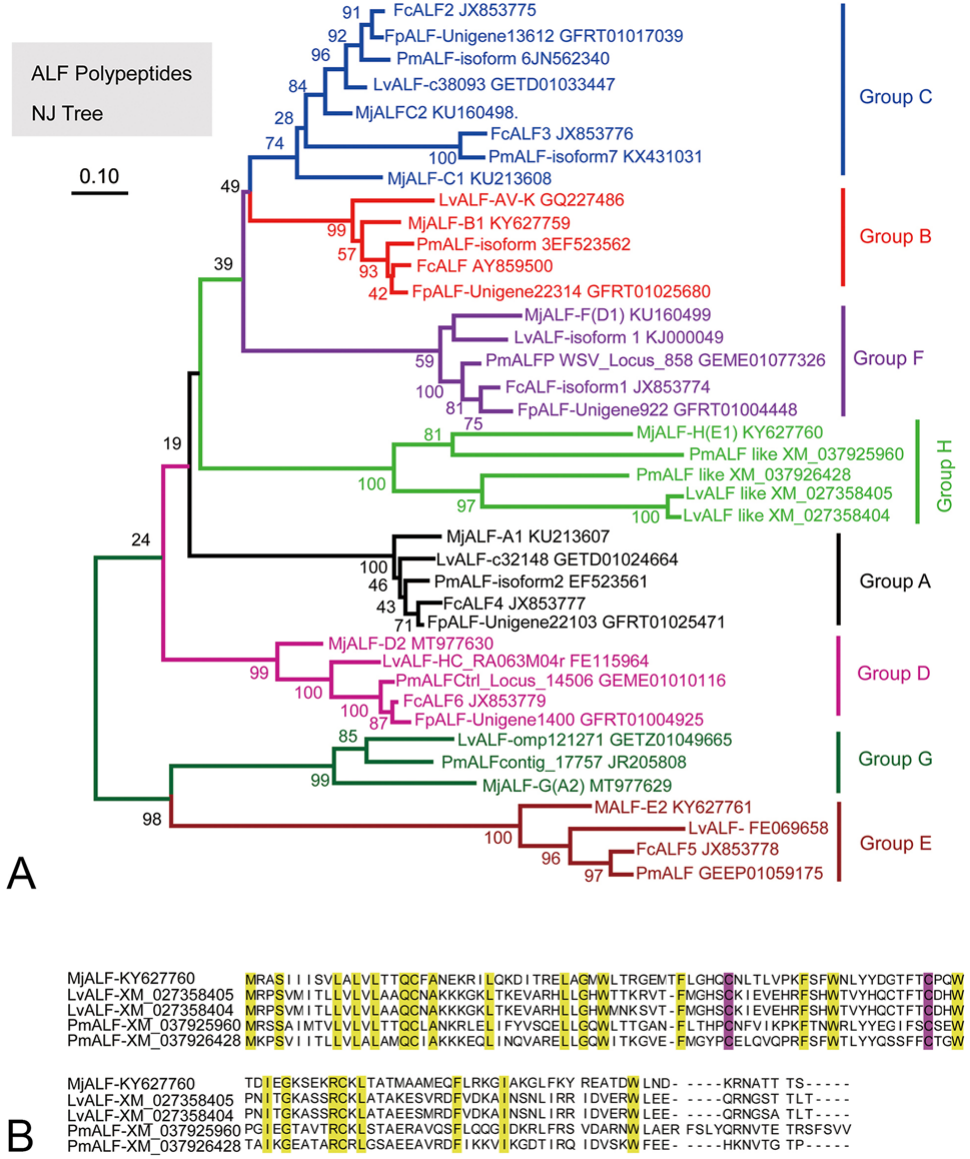
Phylogenetic tree of ALFs and alignment of group ALF-H. **A** Phylogenetic tree of ALFs from penaeid shrimp constructed via MEGA by using neighbor-joining method. Nine ALF members belong to eight groups of the ALF family identified in *M. japonicus*. **B** Alignment of amino acid sequences of five ALFs in group H. Fc, *Fenneropenaeus chinensis*; Fp, *Fenneropenaeus penicillatus*; Lv, *Litopenaeus vannamei*; Mj, *Marsupenaeus japonicus*; Pm, *Penaeus monodon*. The LPS-binding domain is located at two cysteine residues

Nine ALF members were identified in the transcriptomic sequencing of shrimp *M. japonicus*. Then, 29 sequences of ALFs (GenBank accession numbers listed in Fig. 1A) were collected in four other penaeid shrimp species, namely, *F. chinensis*, *F. penicillatus*, *L. vannamei*, and *P. monodon*, from the GenBank database for reconstruction of phylogenetic trees. The members of the ALF family in penaeid shrimp were divided into eight groups (groups A–H, Fig. 1A), and a new group (group H) was identified. These groups could be classified into five clusters, i.e., cluster I (groups C, B, and F), Cluster II (group H), cluster III (group A), cluster IV (group D), and cluster V (groups E and G). The newly identified group H is composed of cationic ALFs with isoelectric points ranging from 9.14 to 9.80 present in *M*. *japonicus*, *L. vannamei*, and *Penaeus monodon* (Fig. 1B). The original MjALF-E1 in the previous report (Jiang et al. 2015) was reclassified as MjALF-H in the reconstructed phylogenetic trees (Fig. 1A). The function of MjALF-E1 was studied, and the results revealed that it could strongly bind to bacteria, and has antibacterial activity (Jiang et al. 2015). The original MjALF-D1 (a cationic peptide) was reclassified as group F (MjALF-F) in the phylogenetic trees (Fig. 1A). In summary, eight groups of ALF family were identified in kuruma shrimp *M. japonicus*, and there has not been any functional study conducted for group D member MjALF-D2.

### Antiviral activity screening using recombinant MjALFs

**Fig. 2:**
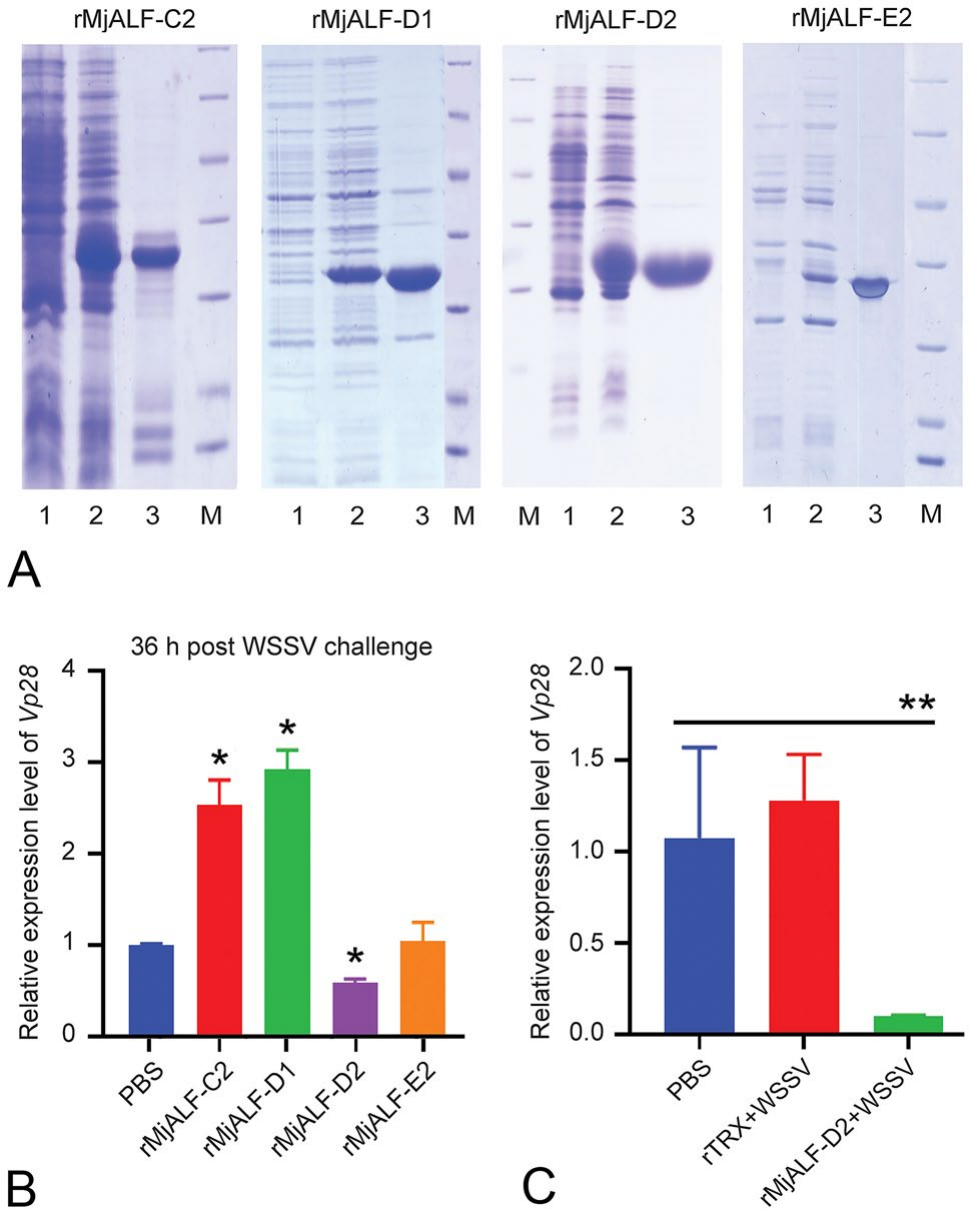
Recombinant expression and purification of four MjALFs and antiviral activity analysis in shrimp. **A** Purification of MjALFs expressed in *E. coli*. and Lane 1, total proteins of *E. coli* with pET32a/*Alf* plasmids without induction; lane 2, total proteins of *E. coli* with pET32a/Alf after IPTG induction; lane 3, purified MjALFs; and lane M, standard protein markers. **B** Expression of *Vp28* in gills of rMjALF-injected shrimp challenged by WSSV, as analyzed by qPCR using *β-Actin* as internal control. **C** qPCR was used to analyze the expression of *Vp28* (encoding the envelope protein of WSSV) in hemocytes of rMjALF-D2-injected shrimp challenged by WSSV. PBS and rTRX injection were used as controls. Asterisks indicate statistical significance (*P < 0.05; **P < 0.01) analyzed by Student’s *t* test

Four recombinant ALF(rALF) polypeptides, including MjALF-C2 (GenBank accession no. KU213608), MjALF-D1 (KU160499), MjALF-D2 (MT977630), and MjALF-E2 (KY627761), were successfully expressed in *Escherichia coli*. After the recombinant ALFs were purified via Ni-NTA chromatography (Fig. 2A), the antiviral effect of the rALFs was screened initially by injecting the rALF and WSSV mixture into shrimp; viral replication was detected at 36 h post injection using the expression of *Vp28* (the envelope protein of WSSV) as the indicator. The results showed that WSSV replication in gills of the rMjALF-D2-injected shrimp was inhibited significantly compared with that in other rALFs and the controls (Fig. 2B). For further confirmation of the results, PBS and thioredoxin (TRX)-tag protein purified from the parent vector pET-32a (+) were used as controls in the antiviral activity analysis. The results showed that compared with PBS and TRX, rMjALF injection significantly inhibited WSSV replication in shrimp hemocytes (Fig. 2C). All the results suggested that MjALF-D2 may possess anti-WSSV function in shrimp.

### *MjAlf-D2* was detected in all tissues tested, and upregulated in shrimp challenged by WSSV

**Fig. 3:**
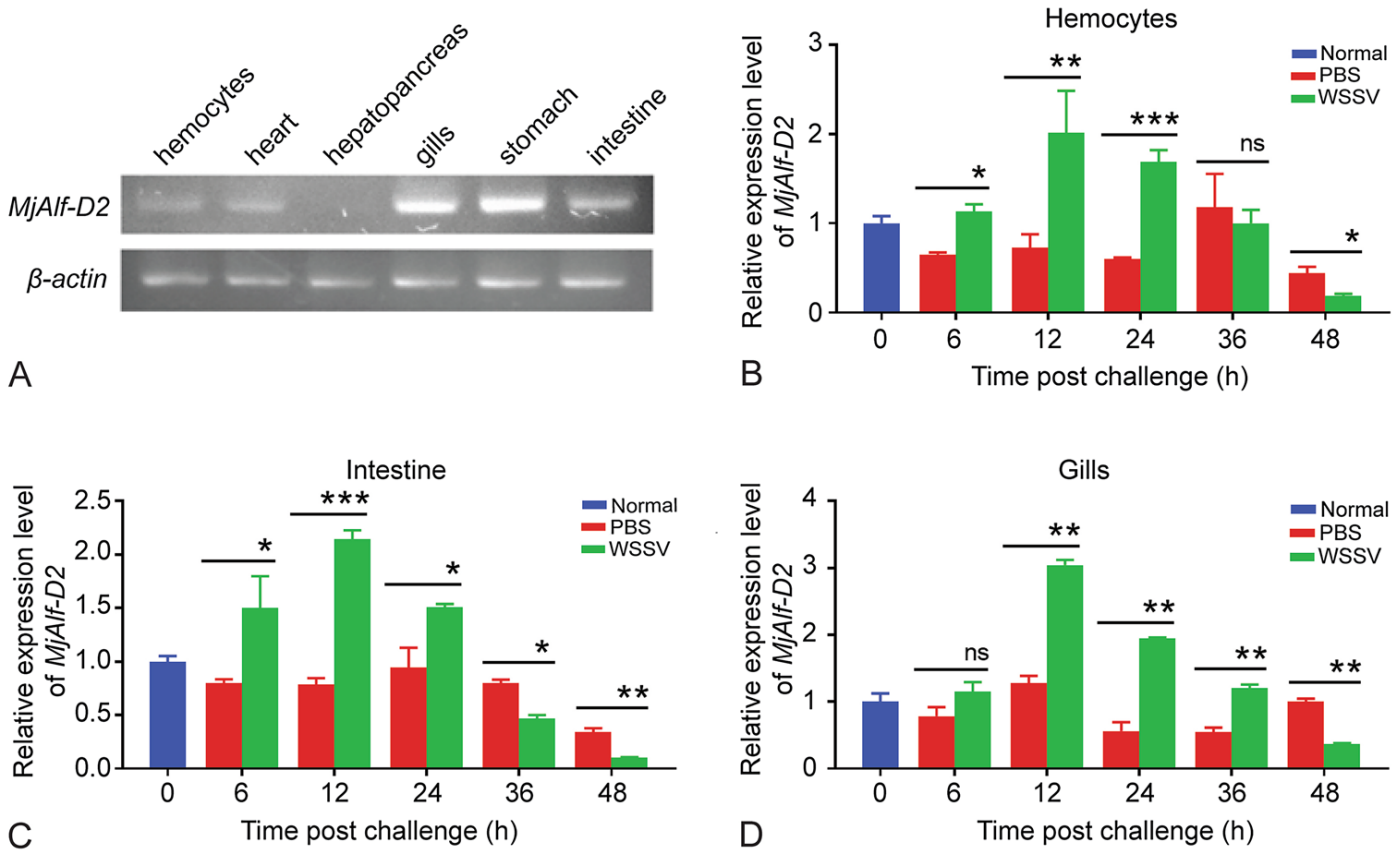
Tissue distribution and expression patterns of *MjAlf-D2* in shrimp. **A** Tissue distribution of *MjAlf-D2* in normal shrimp analyzed by RT-PCR. **B** Expression pattern of *MjAlf-D2* in hemocytes of shrimp challenged by WSSV was analyzed by qPCR. **C** Expression pattern of *MjAlf-D2* in intestine of WSSV-challenged shrimp analyzed by qPCR. **D** Expression pattern of *MjAlf-D2* in gills of shrimp challenged by WSSV. Student’s *t* test was used to analyze statistical significances (*P < 0.05; **P < 0.01; ***P < 0.001)

The tissue distribution of *MjAlf-D2* in the hemocytes, heart, hepatopancreas, gills, stomach, and intestine of untreated shrimp was detected by semi-quantitative reverse transcription polymerase chain reaction (RT-PCR). The results showed that *MjAlf-D2* was detected in all tissues examined, and it was highly expressed in gills, stomach, and intestine (Fig. 3A). The expression patterns of *MjAlf-D2* in different tissues of shrimp challenged by WSSV were analyzed by quantitative real-time PCR (qPCR), and the results showed that after WSSV challenge, *MjAlf-D2* was significantly upregulated in the hemocytes (Fig. 3B), intestine (Fig. 3C), and gills (Fig. 3D) of shrimp. The expression of MjAlf-D2 was upregulated from 6 h, reached the highest level at 12 h, and started to recover from 36 h post WSSV infection. These results suggested that MjALF-D2 was responsible for WSSV infection in shrimp.

### Recombinant MjALF-D2 inhibited the replication of WSSV in vivo

**Fig. 4:**
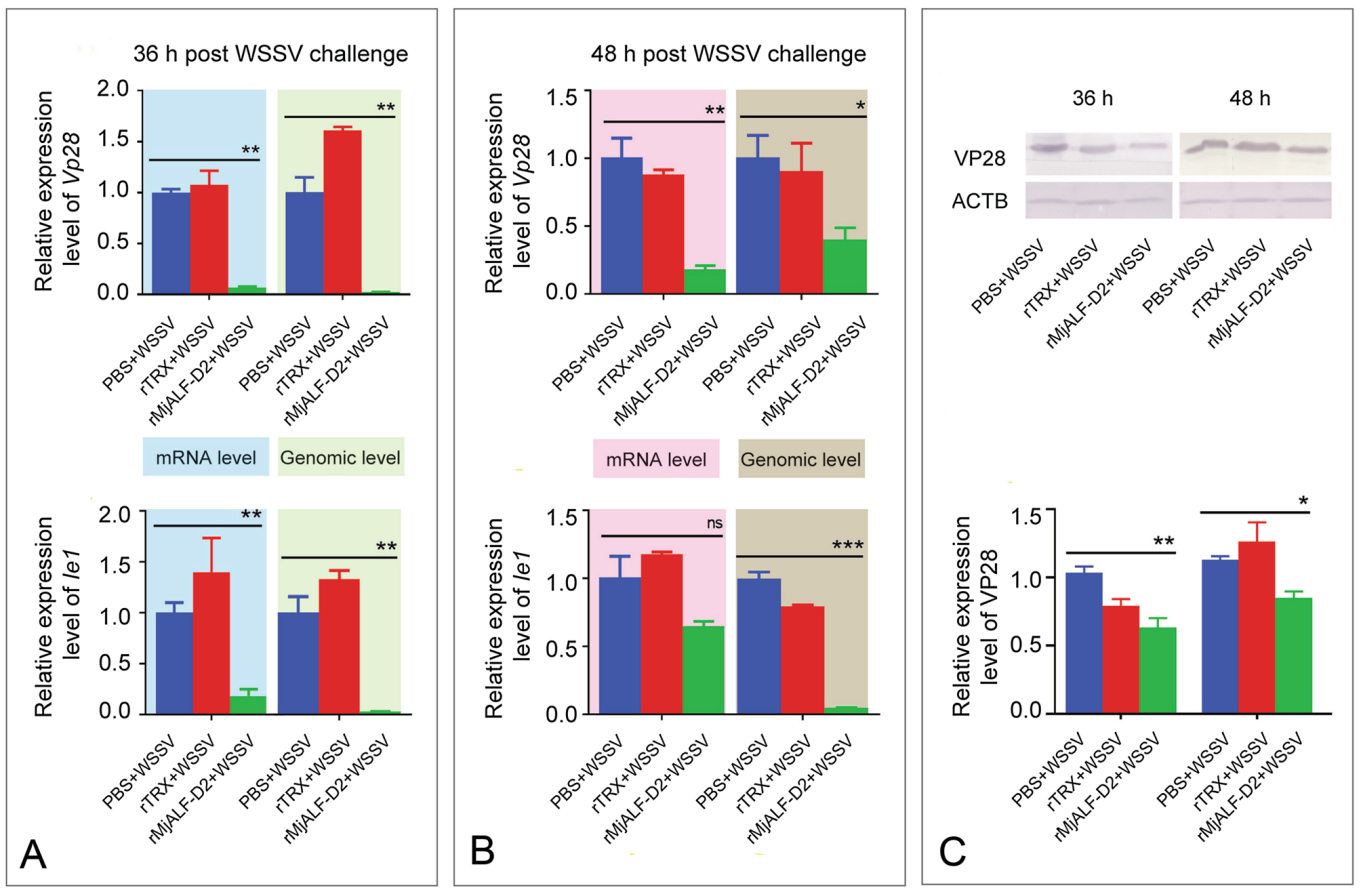
Recombinant MjALF-D2 inhibits WSSV replication in shrimp. **A**, **B** Mixture of rMjALF-D2 and WSSV was injected into shrimp. Expression of *Vp28* (upper panel) and *Ie1* (lower panel) in mRNA and DNA levels was analyzed by qPCR at 36 h (A) and 48 h (B) in gills post WSSV injection. PBS and TRX-tag (rTRX) injection were used as controls. **C** Western blotting was used to analyze the expression of VP28 at protein level in gills of shrimp at 36 and 48 h after WSSV and recombinant protein infection β-Actin (ACTB) was used as loading control. The bands on the membranes of western blot were digitalized using ImageJ software based on three independent repeats (lower panel). The qPCR data were analyzed by Student’s *t* test and asterisks indicated significant difference (*P < 0.05; **P < 0.01; ***P < 0.0001)

WSSV proliferation in RNA, genomic DNA, and protein levels was detected in rMjALF-D2-injected shrimp following WSSV infection, with *Vp28* and *Ie1* (immediate-early gene of WSSV) as indicators, using qPCR to confirm the anti-WSSV activity of rMjALF-D2. The results showed that compared with PBS and TRX injection control, the rMjALF-D2-injected group showed significant reduced expression of *Vp28* and *Ie1* in gills at 36 h (Fig. 4A) and 48 h (Fig. 4B) post WSSV injection. The expression of VP28 in gills of the shrimp was also detected at the protein level by Western blotting, and similar results were obtained (Fig. 4C). These results suggested that rMjALF-D2 inhibits WSSV replication in shrimp.

### VP28 and Ie1 expression increased significantly in MjALF-D2-silenced shrimp

**Fig. 5:**
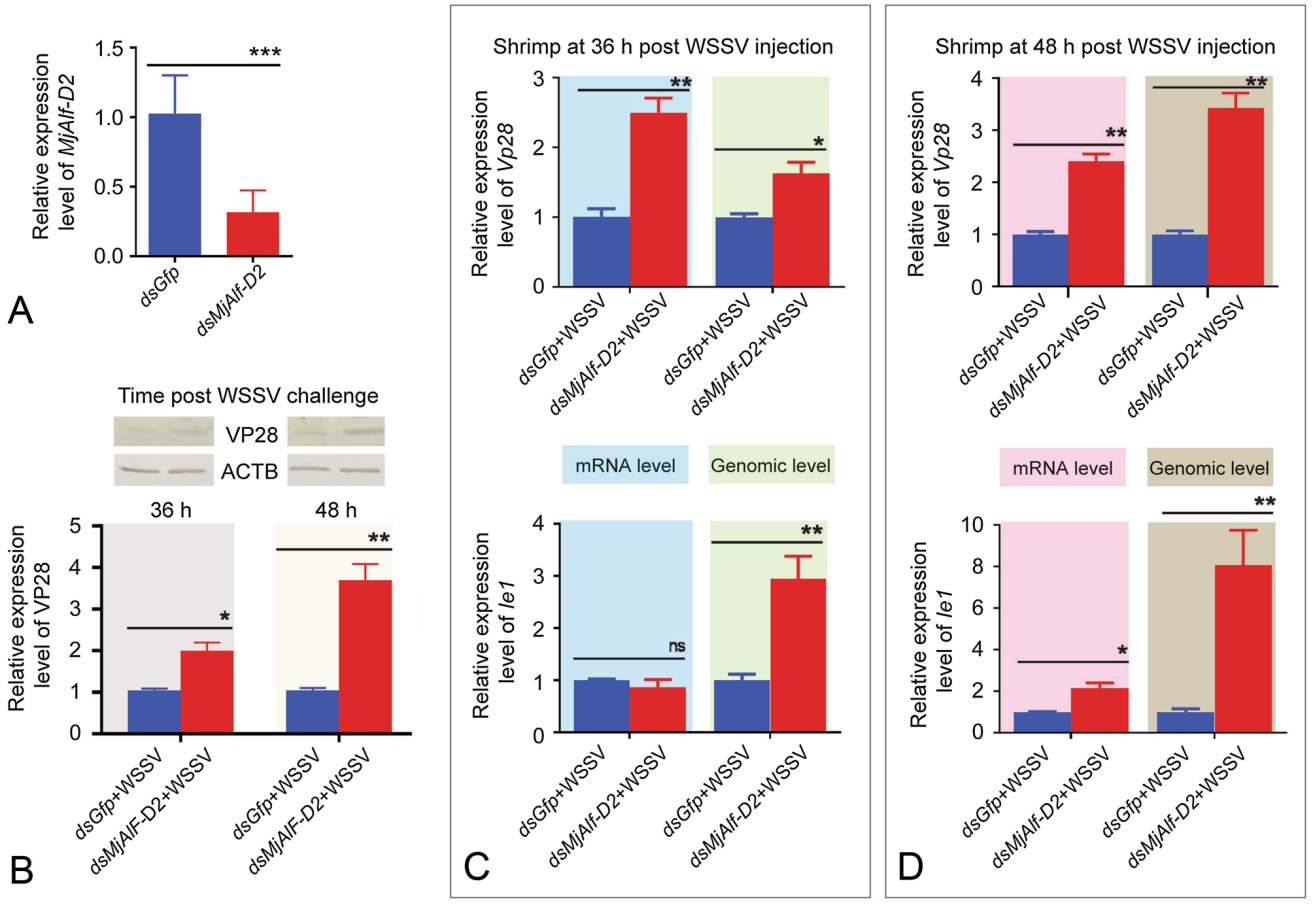
Expression of *Vp28* and *Ie1* increased significantly in *MjAlf-D2*-silenced shrimp. **A** Efficiency analysis of *MjAlf-D2* RNAi in shrimp analyzed by qPCR. **B** Western blotting was performed to analyze the expression of VP28 in gills of *MjAlf-D2*-knockdown shrimp at 36 and 48 h post WSSV challenge. The western blot results were digitalized using ImageJ software based on three independent repeats, and shown in the lower panel of b. **C** Expression of *Vp28* (upper panel) and *Ie1* (lower panel) in mRNA and genomic DNA levels in gills of shrimp at 36 h post WSSV infection analyzed by qPCR. **D** Expression of *Vp28* (upper panel) and *Ie1* (lower panel) in mRNA and genomic DNA levels in gills of shrimp at 48 h post WSSV infection was analyzed by qPCR. Significant differences were indicated by asterisks (*P < 0.05; **P < 0.01) analyzed by Student’s *t* test

*MjAlf-D2* RNAi was performed in vivo to further confirm the role of *Mj*ALF-D2 in shrimp antiviral immunity. *MjAlf-D2* could be knocked down by approximately 65% by the injection of *MjAlf-D2* dsRNA (7 μg/g shrimp) compared with the *dsGfp* injected in the controls (Fig. 5A). The *MjAlf-D2*-silenced shrimp were challenged with WSSV, and the expression of *Vp28* and *Ie1* was detected at mRNA, genomic DNA, and protein levels at 36 and 48 h post WSSV injection. The results showed that the expression of VP28 increased in gills of shrimp at the protein level compared with that of *dsGfp* (Fig. 5B). The expression of *Vp28* and *Ie1* at RNA and genomic DAN levels was also analyzed by qPCR. The results showed that this expression increased significantly in the *MjAlf-D2*-knockdown shrimp challenged by WSSV at 36 h (Fig. 5C) and 48 h (Fig. 5D) post viral injection. All the results suggested that MjALF-D2 exerts important roles in shrimp antiviral immunity.

## Discussion

In this study, the phylogenetic tree of the ALF family from penaeid shrimp was reconstructed, and a new group (group H) was identified. Eight groups of ALF family (A–H) were found simultaneously in kuruma shrimp *M. japonicus*, and the antiviral activity of ALFs in the shrimp was screened using recombinant ALF polypeptides. The anionic ALF-D2 possessed anti-WSSV activity, and this inhibitory activity was further confirmed by RNAi and “overexpression” of MjALF-D2 in shrimp. The results suggested that MjALF-D2 has a potential application for viral disease control in shrimp aquaculture.

Shrimp have an efficient and sophisticated innate immune system that provides protection against invading pathogens. In immune responses, shrimp recognize pathogens via germline-encoded proteins called pattern recognition receptors by sensing pathogen-associated molecular patterns from different micro-organisms (Wang and Wang 2013). Cellular and humoral immunities are initiated in the animals against pathogens by phagocytosis, nodulation, encapsulation, melanization, extracellular traps (Ng et al. 2013) (belonging to cellular responses), or by secretion of AMPs, inhibitors of proteinases, and reactive oxygen species or reactive nitrogen species (belonging to humoral responses) (Bi et al. 2015; Ng et al. 2013; Tassanakajon et al. 2018; Yang et al. 2016a). The AMPs are considered as major humoral immune effectors against invading pathogens by killing or controlling the invading micro-organisms. Among different classes of AMPs, the ALFs are the diverse family of AMP in penaeid shrimp. ALFs exhibit broad-spectrum and powerful antimicrobial activity against major shrimp pathogens. Previous studies identified seven groups of ALFs in *M. japonicus* (Jiang et al. 2015; Matos et al. 2018). Groups A and C were found in a broad range of tissues, including hemocytes, heart, hepatopancreas, gills, stomach, and intestine. ALF-Bs had a high expression level in hemocytes, heart, and intestine. MjALF-D had a low expression in hemocytes, and upregulated by WSSV challenge. MjALF-E had low expression level in hepatopancreas. Group F (originally designated as MjALF-D1) was discovered in heart, gill, stomach, and intestine, and had a high expression in stomach. Group G (originally designated as MjALF-A2, GenBank accession no. MT977629) was mainly distributed in the stomach (Jiang et al. 2015). In the present study, group H was identified as a new group of the ALF family in *M*. *japonicus*, *L*. *vannamei*, and *P*. *monodon*. Eight groups of ALFs were present in a single shrimp, indicating that the different ALFs may act synergistically to improve their antimicrobial activities in vivo. Simultaneous exposure of an invading pathogen to multiple different AMPs may be a critical mechanism for the host effective immune response. A recent study in fruit fly using CRISPR gene editing to knock out all known inducible AMPs showed that AMPs have synergistic, additive, and highly specific microbicidal effects in *Drosophila* (Hanson et al. 2019). A similar situation may exist in penaeid shrimp, such as *M*. *japonicus*.

The antibacterial mechanisms of AMPs are well studied. The bacterial membrane is the main target for most cationic AMPs. Four main models based on interactions between AMPs and bacterial membranes have been proposed: carpet, toroidal pore, aggregate, and barrel-stave models (Jenssen et al. 2006). Some AMPs have activity against enveloped and non-enveloped viruses, and the mechanisms are different based on different AMPs and viruses. A common mechanism of action against enveloped viruses is similar with antibacterial mechanisms, that is, AMPs can destabilize the viral envelope by interaction with the envelope, and damage the virions, thus inhibiting infectivity (Mookherjee et al. 2020). ALFs have direct interaction with the envelope protein of WSSV. For example, the recombinant WSSV189 protein interacts with ALFPm3 (Suraprasit et al. 2014). The antiviral activity of ALFs may be mediated by direct interaction with WSSV proteins. The antiviral mechanism of MjALF-D2 may be similar with that of other AMPs, i.e., it damages the virions by interaction with the viral envelope.

Shrimp aquaculture has grown rapidly and become a major global industry that contributes significantly to socioeconomic development in many countries in Asia and the Americas. However, a succession of bacterial and viral diseases affects the sustainable development of the industry. Shrimp AMPs are small peptides, which are important effectors against a wide spectrum of pathogens in the innate immune response of shrimp. Antibiotics have been used widely in aquaculture, resulting in the development and spread of resistance. Antibiotic resistance has become a global problem due to the increased multidrug-resistant pathogens along with a gradually decline in the discovery of new antibiotics for use in human and animal health (Mookherjee et al. 2020). AMPs are potential alternative of antibiotics as they affect a wide spectrum of pathogens, including viruses. WSSV is the most devastating virus in shrimp aquaculture. It infects all cultured penaeid shrimp species, and causes substantially economic losses globally. The potential use of ALFs in shrimp disease control has been reported previously (Supungul et al. 2017). Administering rALFPm3 could increase shrimp survival after WSSV infection. In the present study, the injection of rMjALF-D2 could inhibit WSSV replication in shrimp, and thus be beneficial to shrimp aquaculture. Therefore, shrimp ALFs have potential therapeutic uses to overcome severe viral disease outbreaks in shrimp aquaculture.

## Materials and methods

### Immune challenge, tissue collection and total RNA extraction

*M. japonicus* shrimp (8–10 g/individual) were bought from the market in Jinan, Shandong Province, China, and cultured as described previously (Niu et al. 2019). The shrimp were acclimated for 48 h in tanks with aerated artificial seawater (salinity between 24 (w/v) and 26) at ~ 24 °C, and randomly taken for use in the following experiments. For pathogen challenge, the shrimp were divided into two groups (30 individuals per group): one group was injected with WSSV (3.2 × 10^5^ copies per shrimp) and the other (control group) was injected with same volume of PBS (Jiang et al. 2013). Hemolymph was collected from five shrimp using sterile syringes with anticoagulant buffer (450 mmol/L NaCl, 10 mmol/L KCl, 10 mmol/L EDTA, 100 mmol/L HEPES, pH 7.45), and then the hemocytes were collected after centrifugation at 800 *g* for 6 min at 4 °C. The other tissues were dissected with scissors and forceps from at least three shrimp, and placed on ice for RNA or protein extraction. The total RNAs from hemocytes and other organs (heart, hepatopancreas, gills, stomach and intestine) of the challenged and control shrimp were extracted using Unizol reagent (Biostar, Shanghai, China). cDNA was synthesized with a pair of primers (SMART F and Oligo anchor R) using a Revert Aid First Strand cDNA synthesis kit (Fermentas, Burlington, Canada).

Before challenge experiments, we randomly took three shrimp to detect if the shrimp are WSSV-free samples using RNA from hemocytes and intestines to analyze the expression of *Vp28* mRNA with specific primers Vp28F and Vp28R by qPCR. The PCR was performed with the following procedures: 95 °C for 10 min; 40 cycles at 95 °C for 10 s and 60 °C for 50 s; and then a melting period from 65 to 95 °C. The data obtained were analyzed using the cycle threshold (2^−∆∆CT^) method. β-Actin was used as the internal control gene.

### Phylogenetic analysis

The full-length coding sequences of MjALFs were obtained from hemocyte-, intestine-, and hepatopancreas-transcriptomic sequencing of *M*. *japonicus* in the laboratory. The ALF full-length sequences of other shrimp were collected from GenBank databases. Neighbor-joining analysis was conducted via MEGA 7, and bootstrap sampling was reiterated 500 times (Kumar et al. 2016).

### Semi-quantitative RT-PCR and quantitative real-time (qPCR) analysis

The tissue distribution of MjALF-D2 in different tissues of non-injected shrimp were analyzed via RT-PCR with specific primers MjAlf RTF and RTR. *β-actin* was amplified as an internal control, with a pair of primers *β-actin* F and *β-actin* R.

qPCR was performed to analyze the transcriptional profiles of *Mj*Alf-D2 at different timepoints after WSSV challenge. The profile of the PCR was 95 °C for 10 min, 40 cycles of 95 °C for 15 s, 60 °C for 60 s, and read at 75 °C for 2 s; melting curve analysis was performed from 68 to 95 °C. The assay was repeated three times, and data were calculated using the 2^–ΔΔCt^ method. Significant differences were analyzed using Student’s *t* test. The figures were created on Graphpad Prism 5 software.

### Expression and purification of four recombinant ALFs

The mature peptide sequences of four MjALFs, including MjALF-C2, MjALF-D1, MjALF-D2, and MjALF-E1, were amplified with different primers (Table 1) via RT-PCR; cloned into pET-32a (+); and transformed into *E. coli* Rosetta cells. The recombinant proteins were purified by affinity chromatography using High-affinity Ni-IDA Resin (Genscript, Nanjing, China). The parent vector pET-32a (+) was used to express the TRX-tag protein in the Rossetta cells. The control protein (TRX) was also purified using the same methods described above with High-affinity Ni-IDA Resin (Genscript, Nanjing, China).

**Table 1:** Primers used in the study

Primer	Sequence (5′-3′)	Direction
β-actinF	AGTAGCCGCCCTGGTTGTAGAC	Forward
β-actinR	TTCTCCATGTCGTCCCAGT	Reverse
MjALF-D2RNAi-F	GCGTAATACGACTCACTATAGGAGCACCTACAGTCATCAC	Forward
MjALF-D2RNAi-R	GCGTAATACGACTCACTATAGGAATCTCAAAGTTATTCTA	Reverse
Gfp RNAi-F	TAATACGACTCACTATAGGGGGGTGGTCCCAATTCTCGTGGAAC	Forward
Gfp RNAi-R	TAATACGACTCACTATAGGGCTTGTACAGCTCGTCCATGC	Reverse
MjAlf-D2RT-F	CGCAGGCTTATGGAGGAC	Forward
MjAlf-D2RT-R	AGGTGACAGTGCCGAGGA	Reverse
MjAlf-C2EXF	TACTCAGAATTCCAGGGGTGGGAGGCACTCGTGCCA	Forward
MjAlf-C2EXR	TACTCACTCGAG TTACTGATTTAACCAAGCCT	Reverse
MjAlf-D1EXF	TACTCAGAATTCCAAATATGGGAGACGCTGAT	Forward
MjAlf-D1EXR	TACTCACTCGAGTTACTTGTTGAGCCACGCCT	Reverse
MjAlf-D2EXF	TACTCAGAATTCCAGGGACTAAAGGACTTTTTAT	Forward
MjAlf-D2EXR	TACTCACTCGAGCTACACGATATATGGTTTTG	Reverse
MjAlf-E2EXF	TACTCAGAATTCATGATGACGTCACCCAATCC	Forward
MjAlf-E2EXR	TACTCACTCGAGTTACAGCCACTCTGCCGCTT	Reverse
Vp28F	AGCTCCAACACCTCCTCCTTCA	Forward
Vp28R	TTACTCGGTCTCAGTGCCAGA	Reverse
Ie1F	GACTCTACAAATCTCTTTGCCA	Forward
Ie1R	CTACCTTTGCACCAATTGCTAG	Reverse

### Antivirus activity analysis of recombinant ALFs

WSSV inoculation was prepared following the previous method with a few modifications (Yang et al. 2016b). The gills (1 g) of WSSV-infected crayfish *Procambarus clarkii* were homogenized in 10 ml PBS (140 mmol/L NaCl, 2.7 mmol/L KCl, 10 mmol/L Na_2_HPO_4_, and 1.8 mmol/L KH_2_PO_4_; pH 7.4), and then centrifuged at 5000 *g* for 10 min at 4 °C. The supernatant was filtered through a 0.45 μm filter (Jinlong, Tianjin, China). The viral titer was determined via qPCR on the basis of a previous report (Wang et al. 2014b). *M. japonicus* were divided into three groups, and the shrimp in each group was injected with PBS, TRX, or *Mj*ALFs (30 µg). One hour later, the shrimp was challenged with WSSV (2.5 × 10^5^ copies). Genomic DNA, mRNA, and proteins were extracted from the gills of WSSV-infected and control shrimp at 36 and 48 h post WSSV injection. The genomic DNA was extracted using the MagEtractor Genomic DNA purification kit (Toyobo, Shanghai, China), and WSSV replication was analyzed using qPCR, with *Vp28*, a WSSV envelope protein, as an indicator.

### RNA interference assay

The dsRNAs of MjALF-D2 or green fluorescent protein (GFP) were synthesized using the DNA template amplified with specific primers *Mj*Alf-D2RNAiF, RNAiR, GfpF, and GfpR linked to the T7 promoter (Table 1) by T7 RNA polymerase (Fermentas, Thermo Fisher Scientific, USA). The shrimp were randomly divided into two groups. Each shrimp in the two groups was injected with 30 μg of *dsMjAlf-D2* or *dsGfp* two times every 24 h. Total RNA was extracted from the gills of the shrimp at 48 h post second injection using TRIzol (TransGen, Beijing, China) to detect the RNAi efficiency. Then, WSSV (2.5 × 10^5^ copies) was injected into the *MjAlf-D2*-silenced shrimp at 48 h post second *dsRNA* injection. Genomic DNA, mRNA, and proteins were extracted from the gills of the shrimp at 36 and 48 h post WSSV injection using the method described above for the detection of WSSV replication.

### Western blotting

Western blotting was used to detect WSSV replication by using VP28 antibody. The gills of *MjAlf*-*D2*-RNAi shrimp were homogenized in the buffer (50 mmol/L Tris–HCl, 150 mmol/L NaCl, 1 mmol/L EDTA, and 1 mmol/L phenylmethylsulfonyl fluoride; pH 7.5), and the homogenate was centrifuged at 10,000 *g* for 10 min at 4 ℃. The proteins of the sample were separated by 12.5% SDS–polyacrylamide gel electrophoresis and transferred onto nitrocellulose membranes. The membranes were blocked with 3% nonfat milk in TBS (10 mmol/L Tris–HCl and 150 mmol/L NaCl; pH 7.5) for 1 h, and incubated for 2 h with 1/100 diluted antiserum against VP28 in TBS with 3% nonfat milk. Then, after washing to remove the free, nonspecifically binding antiserum, horseradish peroxidase-conjugated goat anti-rabbit IgG (1:10,000 dilution in TBS) was added and incubated for 2 h. The membrane was put in the reaction system (10 ml of TBS with 45 μl of NBT and 35 μl of BCIP) in the dark for 5 min to visualize the signal. The antibody against the VP28 and β-actin used in Western blotting was prepared in the laboratory. The bands on the membrane of western blot of three independent repeats were digitalized by scanning using ImageJ software.

### Statistical analysis

Data are presented as mean ± SD (*n* = 3). Statistical evaluation of significant differences between experimental groups was conducted using Student’s *t* test. The *P* values are defined as follows: *P < 0.05; **P < 0.01; ***P < 0.001.
